# Effects of the Moral Treatment of Hysterical Fits

**Published:** 1850-04

**Authors:** 


### OBSTETRICS.

*Effects of the Moral Treatment of Hysterical Fits.*—A young lady, who had met with a very severe disappointment, was placed under our care. She was 23 years of age, and hereditarily predisposed to the disease on both sides. It manifested itself by an excited state of mind, with startings and restlessness, and she had frequent hysterical fits. Every attention had been paid to her health before she quitted home, but having attempted to throw herself out of the window, it was deemed proper to remove her from the scene of her excitement. She was cheerful and clever, and very susceptible of admiration. When she first came, she stated that her fits were so frequent that it was not right for her to go to church; but as they were really not violent, we observed to her that it was always the rule of the house to go to church, and that if the fit came on there, we should be obliged to call for the assistance of the beadle to take her out; and that she would thus make herself very conspicuous. After the service of the first Sunday, she observed, on coming home from church, that she was very nearly attacked indeed; and it was remarkable that she never had any fit at these times afterwards. We took courage from this, and hoped the time would come when the fits would disappear, not only at church, but altogether, by a similar method of treatment. One day, while at dinner, her knife and fork dropped suddenly into her plate, and she was simultaneously upon the floor. There were several at the table, and the servant was requested to give no heed to the lady. After a few minutes had passed away, a gentleman who was at the table, whose pharmaceutical knowledge would never make his fortune, feeling a little nervous about the issue of the case, rather anxiously suggested that she should have some *Epsom* salts—meaning, no doubt, to say smelling salts, given to her. This was quite enough. She laughed very much, and resumed her place at the table, and all went on as before. And it it is very pleasing to be able to add, that for the few months longer she remained with us she experienced no return of the fits either at church or at home; her irritability and oddness of manner went off, and she continued well. This is now eighteen years ago, and there has been no actual return of the threatened malady, though she has been extremely nervous at times, many sorrows and trials having attended her. If these fits had been neglected or encouraged by bad management at the first, the probability is, she would, with all the predisposing circumstances of the case, have been the subject of insanity at the present time.—*Dr. Burnett on Insanity.*

